# Gene Regulation of Intestinal Porcine Epithelial Cells IPEC-J2 Is Dependent on the Site of Deoxynivalenol Toxicological Action

**DOI:** 10.1371/journal.pone.0034136

**Published:** 2012-04-10

**Authors:** Anne-Kathrin Diesing, Constanze Nossol, Siriluck Ponsuksili, Klaus Wimmers, Jeannette Kluess, Nicole Walk, Andreas Post, Hermann-Josef Rothkötter, Stefan Kahlert

**Affiliations:** 1 Institute of Anatomy, Otto-von-Guericke University Magdeburg, Magdeburg, Germany; 2 Leibniz Institute for Farm Animal Biology (FBN), Molecular Biology, Dummerstorf, Germany; University of Pittsburgh, United States of America

## Abstract

The intestinal epithelial cell layer represents the border between the luminal and systemic side of the gut. The decision between absorption and exclusion of substances is the quintessential function of the gut and varies along the gut axis. Consequently, potentially toxic substances may reach the basolateral domain of the epithelial cell layer via blood stream. The mycotoxin deoxynivalenol (DON) is a *Fusarium* derived secondary metabolite known to enter the blood stream and displaying a striking toxicity on the basolateral side of polarised epithelial cell layers *in vitro*. Here we analysed potential mechanisms of apical and basolateral DON toxicity reflected in the gene expression. We used the jejunum-derived, polarised intestinal porcine epithelial cell line IPEC-J2 as an *in vitro* cell culture model. Luminal and systemic DON challenge of the epithelial cell layer was mimicked by a DON application from the apical or basolateral compartment of membrane inserts for 72 h. We compared the genome-wide gene expression of untreated and DON-treated IPEC-J2 cells with the GeneChip® Porcine Genome Array of Affymetrix. Low basolateral DON (200 ng/mL) application triggered 10 times more gene transcripts in comparison to the corresponding apical application (2539 versus 267) despite the intactness of the challenged cell layer as measured by transepithelial electrical resistance. Analysis of the regulated genes by bioinformatic resource DAVID identified several groups of biochemical pathways modulated by concentration and orientation of DON application. Selected genes representing pathways of the cellular metabolism, information processing and structural design were analysed in detail by quantitative PCR. Our findings clearly show that apical and basolateral challenge of epithelial cell layers trigger different gene response profiles paralleled with a higher susceptibility towards basolateral challenge. The evaluation of toxicological potentials of mycotoxins should take this difference in gene regulation dependent on route of application into account.

## Introduction

The intestinal mucosa represents the border between the organism and the environment and is the first barrier to nutrients and other, potentially harmful, substances such as mycotoxins. A polarised epithelial cell architecture is necessary to meet the demands of these functions. The luminal side of the intestinal epithelium is organised in villi, crypts and microvilli to enhance the surface and further covered with protective complex mucus. Epithelial cells express intercellular connections known as tight junctions, which form a largely impermeable barrier to prevent unspecific penetration of molecules and microorganisms into the body. The basolateral side of the epithelial cell layer is facing the porous basal lamina and is, via the underlying *lamina propria*, in contact with cells and components of the blood circulation. As a consequence epithelial cells can be challenged from both the apical as well as the basolateral compartment.

Mycotoxin contamination of crops is a serious problem in animal farming, especially for pigs as they are identified as the most susceptible species to this mycotoxin. DON affects growth and function of intestinal epithelial cells by several pathological mechanisms, including activation of cellular signalling and ribosomal stress. In principle, DON can interact with epithelial cells on the apical side during intestinal passage and absorption. It has been shown that principal DON absorption occurs in the porcine stomach and upper small intestine. However, detectable concentrations of DON were found in blood serum and it can be assumed, that epithelial cells of the small intestine and the colon are also exposed to DON via blood stream from the basolateral side [1].

As recently shown, IPEC-J2 is a promising porcine epithelial cell culture model which retained most of its original epithelial nature [2]. Cultured on membrane inserts, epithelial structures such as microvilli and tight junctions occur [3]. This approach allows the analysis of the effects of both apical and basolateral application of low and high DON concentrations. In preceding investigations we found a clear dependency between DON toxicity and the side of DON application *in vitro* [4]. Recent advances in transcriptomics in swine have opened new opportunities for a global survey on the genetic background of complex traits [5].

Consequently, we employed comparative, genome-wide expression profiling of polarised IPEC-J2 cell layers in response to side specific DON treatment. The objective of this investigation was to detect potential expression differences and, subsequently, to identify candidate genes for further investigations on cellular reaction mechanisms to DON exposure in intestinal epithelial cells.

## Materials and Methods

### 1. Cell culture conditions

IPEC-J2 cells (ACC701, DSMZ), representing non-transformed, polarised-growing intestinal porcine epithelial cells [6], continuously maintained in cell culture, were used in this study [7]. Cells were cultured in Dulbecco's modified eagle medium (DMEM/Ham's F-12 (1∶1)) supplemented with 5% fetal calf serum (FCS), 1% insulin-transferrin-selenium (ITS), 16 mmol/L HEPES (all PAN-Biotech, Germany) and 5 ng/mL epidermal growth factor (EGF; BD Biosciences, Germany) and incubated at 39°C and 5% CO_2_ [3]. The cell culture was regularly tested and found to be free of mycoplasma contamination (Venor® GeM Mycoplasma Detection Kit; Minerva Biolabs, Germany). The cells were routinely seeded at a density of 0.5×10^5^/mL with 7.5 mL medium in plastic tissue culture flasks (25 cm^2^ Nunc, Denmark) and passaged every 3–4 d for a maximum of 20 times (passages 78–98). For the use in experiments, cells were seeded at a density of 2.0×10^5^/well (membrane area 4.5 cm^2^/well) in uncoated 6-well, 1 µm pore-sized ThinCert™ membrane inserts (Greiner bio-one, Germany) and cultured for 7 d.

### 2. Transepithelial electrical resistance (TEER)

TEER measurements were performed using a Millicell Electrical resistance system (Millipore, France). Cells were determined to be confluent at a TEER value of >1 kOhm/well, corresponding to 4.5 kOhm/cm^2^, indicating an intact, tight junction connected monolayer, which was usually measured after 7 d of incubation.

### 3. Preparation of deoxynivalenol (DON)

DON (D0156; Sigma-Aldrich, Germany) was diluted in absolute ethanol (99.6%; Roth, Germany) to a 0.2 mg/mL stock solution and working dilutions were prepared in cell culture medium. A low DON concentration of 200 ng/mL and a high DON concentration of 2000 ng/mL were applied reflecting a non-toxic and a toxic dosage as found in conventional toxicological studies [8]. A final concentration of 1% ethanol corresponding to the ethanol concentration of 2000 ng/mL DON solution was tested and results were not significantly different from control [8].

### 4. Sample collection and extraction of total RNA

After 72 h of incubation with DON either from apical or basolateral side, cells were washed with PBS and collected from membranes with a cell scraper. Total RNA was isolated using TRIzol Reagent (Invitrogen, Germany) according to the manufacturer's protocol. Briefly, cells were lysed, chloroform added and RNA was recovered from the aqueous phase by precipitation with isopropyl alcohol, dried and dissolved in DEPC water (Roche, Germany). After DNaseI treatment RNA was cleaned up with the RNeasy® Kit (Qiagen, Germany). The quantity of RNA was established using the NanoDrop ND-1000 spectrophotometer (Peqlab, Germany) and the integrity was checked by running 1 µg of RNA on a 1% agarose gel. The RNA samples were stored at −70°C until processing.

### 5. Array analysis

Intestinal epithelial cell expression patterns were assessed using the GeneChip® Porcine Genome Array (Affymetrix, UK). This array contains 24,123 probe sets representing transcripts from 20,201 *Sus scrofa* genes. Tsai et al. [9] improved the annotation of the array by assigning approximately 82% of the transcripts to 11,265 different porcine genes. The fragmentation and labelling was performed with the GeneChip® Terminal Labeling Kit (Affymetrix, UK) according to the manufacturer's recommendations. A total amount of 500 ng RNA per sample was used for preparation of antisense, biotinylated RNA for hybridization. After hybridization, washing and scanning of the arrays, primary data analysis with Affymetrix GCOS 1.3 software was performed using global scaling to a target signal of 500. The raw data files were provided along with a summary of the analysis containing probe set identification, quality measures for the hybridization, the relative expression value and a qualitative measure for the probe sets (present, absent or marginal) for each individual array.

### 6. Array data analysis

Bioinformatic analysis of the microarray data was performed in 3 steps: (A) quality control of all arrays, (B) preprocessing of all arrays (background correction, normalization, summary measures for probe sets), and (C) identification of differently expressed genes.

The quality of hybridization was assessed in all samples using Affymetrix Expression Console software (Affymetrix, St. Clara, USA). First, the data were processed with the MAS5.0 algorithm to generate probe cell intensity values, i.e. single expression value for each probe set that are derived from intensities of pairs of perfect-match probes and mismatch probes, and to evaluate presence and absence of transcripts. Using default settings (detection p-values of <0.04 for ‘present’, ≥0.04 and ≤0.06 for ‘marginal’, and <0.06 for ‘absent’) only ‘present’ calls were used. The subsequent data processing, including background correction, probe summarization and normalization, was performed using the probe logarithmic intensity error (PLIER) algorithm that reveals summary values for the probe sets. The microarray data related to all samples were deposited in the Gene Expression Omnibus public repository (GEO accession number).

After quality control and background correction all arrays could be used for further analysis. Affymetrix IDs were mapped to the corresponding gene symbols based on the assignments available from the Ensembl database (http://www.ensembl.org), and mean values over all corresponding Affymetrix IDs were calculated. Because pairs of “Control” and “DON-treated” cell cultures are full siblings, a paired t-test was used to assess statistical significance of differentially expressed genes (p<0.05). The resulting lists for each of the four DON-treated cell cultures from three independent experiments were compared with control to identify regulated genes. Transcript levels significantly above control levels of untreated cells were designated as “up-regulated” and control levels significantly below untreated control levels were designated as “down-regulated”. Data were subsequently analysed with different options of the DAVID Bioinformatic resources [10] for the identification of functional pathways in the different experimental groups. Biological pathways were designated according to the Kyoto Encyclopedia of Genes and Genome (KEGG) database (http://www.genome.jp/kegg/).

### 7. Quantitative PCR

Quantitative PCR (qPCR) was performed on 11 genes identified from the analysis of the gene expression data as potential candidate genes for key mechanisms regulated by DON-treatment. The primers were designed from the same porcine expressed sequence tags (EST) used for the development of the respective probe sets on the Affymetrics GeneChip® Porcine Genome Array (Table 1).

**Table 1:** Primer sequences for the genes analysed with qPCR.

Gene symbol	Gene name	Primer sequence left (5′–3′)/right (5′–3′)	Product in bp	Annealing temperature in °C	Efficiency in %
CTNNB1	ß-Catenin	CCCGAATTGACAAAATTGCT/TGCAGACACCATCTGAGGAG	132	56.1	99.3
CLDN3	Claudin-3	GTCCATGGGCCTGGAGAT/GATCTGCGCTGTGATAATGC	130	58.7	99.5
CYC1	Cytochrome C-1	CTACCATGTCCCAGGTAGCC/AAAGCAAGCCCATCATCATC	117	54.7	96.7
SDHB	Succinate dehydrogenase	ACTGGATGGGCTGTACGAGT/GTCGATCATCCAGCGATAGG	127	55.7	96.7
PDHA1	Pyruvate dehydrogenase	ACCCGATCATGCTTCTCAAG/TAGCAAACTGTGCAGCATCC	117	54.0	96.7
LAMP2	Lysosome associated membrane glycoprotein 2	TTTGGTTCTGAGTGTTTTTCATGT/AATCAAACCCAGGCCACAG	120	54.0	96.7
LIG1	DNA ligase 1	CTTCGCTTCCCTCGGTTTAT/GTGCCTTGCTGGTTCTGAAT	119	54.0	100.2
CDKN1A	Cyclin dependent kinase inhibitor 1 (p21)	TCATTGCACTTTGAACAGCAG/TCCGGAAAGACAACAACTCC	129	54.0	99.3
RPL10a	Ribosomal protein 10a	TGCCTTTTTGGCTTCAGAAT/ACTTTGGCCACCATGTTCTC	120	54.5	99.0
TRA1	Endoplasmin precursor (GRP94)	CAGACACTGGTGTGGGAATG/TGACTGGCCATCTTCTTGTG	119	54.0	105.3
CAV2	Caveolin-2	TGCCTTAGGCCATTGTAACAT/AAGTTGTGAGACTGATTGACCTTTT	124	54.0	98.9
ACTB	ß-Actin	GATGAGATTGGCATGGCTTT/CACCTTCACCGTTCCAGTTT	122	54.0–58.7	99.0–100.2

Annealing temperature and corresponding efficiency are indicated.

RNA obtained from five independently repeated experiments were used as template for qPCR. Each 1 µg of template RNA was subjected to reverse transcription with First Aid Reverse Transcription Reagents (Fermentas, Germany) essentially as described by the manufacturer with the supplied random hexamer primers in a ThermalCycler TC1 (Biometra, Germany). The resulting cDNA samples were used for qPCR amplification of the 11 chosen genes.

Quantitative PCR amplification was performed for all genes under following conditions on an iCycler (BioRad, Germany): 1.5 min at 95°C, 5 min at 95°C followed by 40 cycles of 30 s at 95°C and 60 s at optimal primer annealing temperature (Table 1). Melting curve analysis (50–95°C) was used for assessing amplification specificity. The reaction volume of 25 µL contained 12.5 µL Maxima Mastermix (Fermentas, Germany) with SYBR® Green and Fluorescein as internal standard, 300 nM of the respective primers (2.5 µL each), 0.5 µL UNG (Uracil-DNA-glycosilase), 6.5 µL nuclease free water and 1 µL cDNA (60 mg/mL).

Analysis of the expression data was done according to the relative standard curve method. A standard curve was derived for each single gene from a serial dilution of the cDNA. The analysis comprised five independent experiments with each sample in triplicate. The ddCt method [11] was used for the calculation of differences in the gene expression (ratio = 2^−ddCt^). The differences between the DON-treated samples and the untreated control in the relative quantification (rq) values were normalized to the individual expression of the housekeeping genes ß-actin and GAPDH. Both ß-actin and GAPDH levels were not significantly influenced by any DON treatment (results not shown).

### 8. Statistical analysis

Results are expressed as means with SEM of three independent experiments for microarray and five experiments for qPCR. Statistical analysis of qPCR was performed using ANOVA and Dunnett's post-hoc test. Asterisks indicate significant differences from untreated control of IPEC-J2 (* p≤0.05; ** p≤0.01)

## Results

### 1. Barrier integrity of cell layer after DON treatment

**Figure 1:**
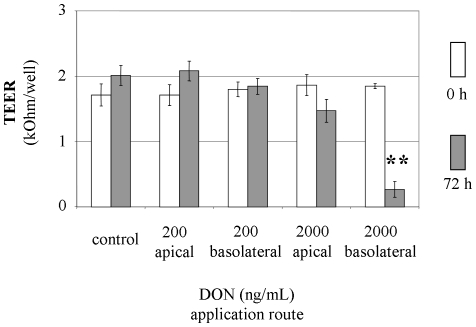
Response of transepithelial electrical resistance (TEER) to apical and basolateral deoxynivalenol (DON) application in polarised IPEC-J2 cells. Cells were grown for 7 d on membrane inserts (0 h) and incubated than for 72 h with DON (0, 200 or 2000 ng/mL) from apical or basolateral side. TEER values are given as kOhm per insert (membrane area 4.5 cm^2^) with 1 kOhm being the level of confluence. Mean±SEM from 5 separate experiments. Significant differences to untreated control were calculated by ANOVA and Dunnett's post hoc test (** p≤0.01).

Integrity of the IPEC-J2 epithelial cell layer *in vitro* was monitored by the measurement of TEER after 7 d of initial cultivation and membrane cultures with a TEER >1 kOhm were included in the experiment. DON in a concentration of 200 ng/mL and 2000 ng/mL was applied to the apical or basolateral compartment, respectively, and incubated for 72 h (Fig. 1). Significant reduction of TEER was found as a consequence of basolateral application of 2000 ng/mL DON only. The TEER of the epithelial cell layer was insensitive to an apical application of the same DON concentration. Application of low DON dosage (200 ng/mL) on the apical or basolateral side of the epithelial cell layer did not significantly influence TEER.

### 2. Gene expression microarray and qPCR analysis

**Figure 2:**
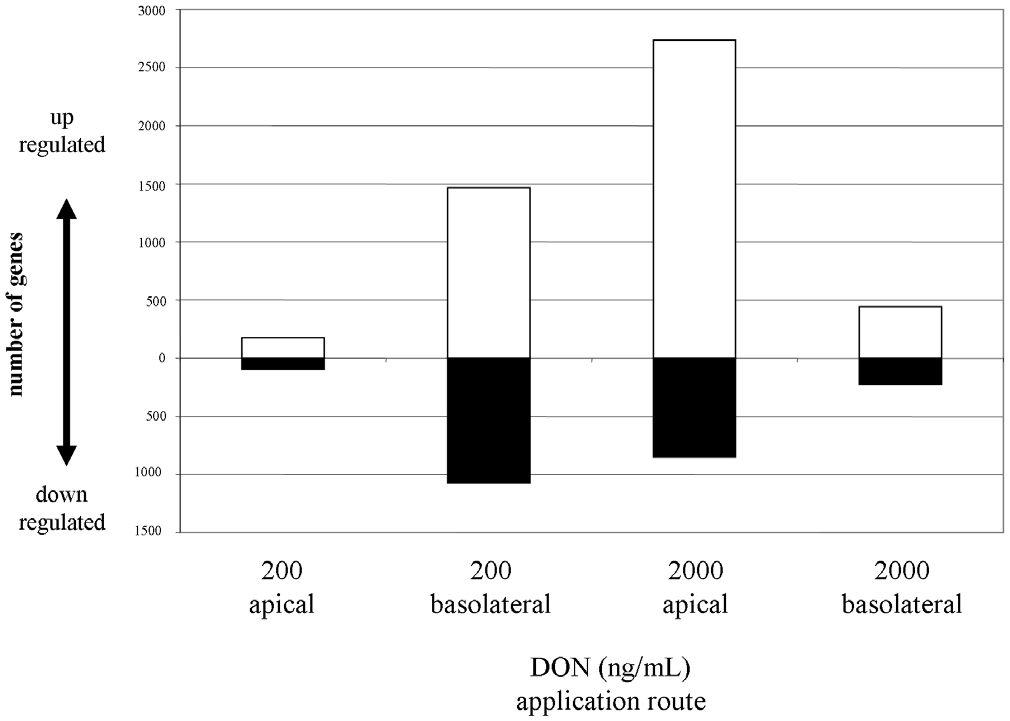
Number of regulated IPEC-J2 genes after 72 h apical and basolateral DON treatment analysed by microarray. Membrane cultures were treated with 200 and 2000 ng/mL DON from apical and basolateral side for 72 h. Isolated mRNA was analysed with GeneChip® Porcine Genome Array. Number of significantly (p<0.05) up- or down-regulated genes in comparison to untreated control genes are given. Values represent numbers of annotated and non-annotated genes of three independent experiments.

The microarray analysis of RNA obtained from DON-treated IPEC-J2 membrane cultures exhibited a response pattern different from that detected with TEER measurements. In Fig. 2 the number of up- or down-regulated transcripts is given in comparison to untreated control. The values represent both annotated and non-annotated genes. In contrast to the physical barrier function as measured by TEER the strongest effects in gene expression were found with 200 ng/mL DON basolateral (2539 changed genes) and 2000 ng/mL DON apical (3589 changed genes). In both conditions TEER was not significantly reduced in comparison to control. Application of 200 ng/mL DON on the apical side caused 10 times fewer changes (267 changed genes) than basolateral application and had no effect on TEER. Interestingly, the significant reduction of TEER, indicating the breakdown of epithelial barrier found with 2000 ng/mL DON from basolateral side, was not accompanied by a strong change in gene expression (669 changed genes).

**Figure 3:**
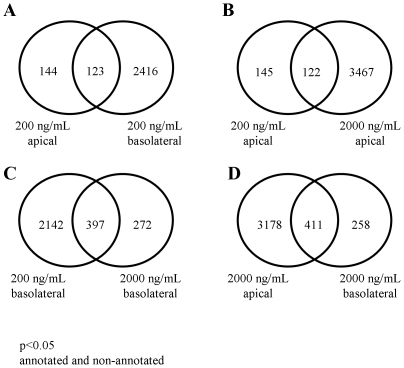
Genes commonly regulated in different treatment groups (Venn diagram). Number of genes commonly regulated in response to (**A**) 200 ng/mL apical or basolateral DON application, (**B**) 200 or 2000 ng/mL apical DON application, (**C**) 200 or 2000 ng/mL basolateral DON application and (**D**) 2000 ng/mL apical or basolateral DON application.

We analysed the degree of overlapping between the groups. Nearly half of the significantly regulated genes (p<0.05) of 200 ng/mL DON apical (123 of 267) were also found in basolateral application of this concentration (Fig. 3A). A similar pattern was found when the apical concentration was 10 times increased. 122 of 267 significantly regulated genes of the 200 ng/mL DON apical condition were also found with 2000 ng/mL DON apical (Fig. 3B). The comparison of the 200 ng/mL and 2000 ng/mL DON application on the basolateral side resulted in 397 shared genes, representing 59% of the regulated genes in the 2000 ng/mL DON basolateral condition (Fig. 3C). The overlap between 2000 ng/mL DON apical and basolateral showed that 61% of the regulated genes of the basolateral application were also found in response to the apical application of DON (Fig. 3D). The application of 200 ng/mL DON from basolateral side of the epithelial layer triggered 10 times more genes than from apical side, whereas the increase of basolateral DON concentration reduced the numbers of regulated genes.

**Figure 4:**
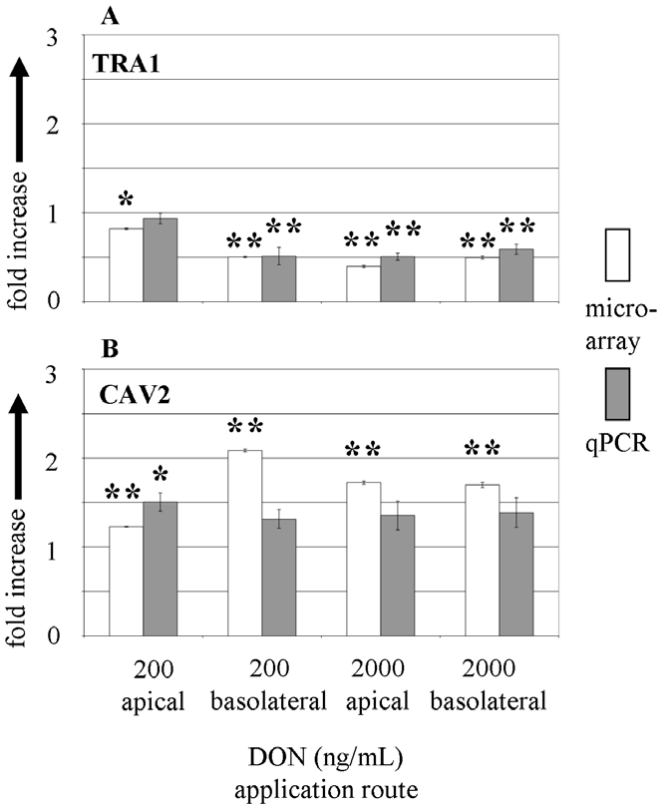
Expression of selected genes measured by microarray and qPCR in membrane cultured IPEC-J2 cells regulated in all DON treatments. Regulation of transcripts of (**A**) TRA1 and (**B**) CAV2 in response to apical and basolateral DON (200 and 2000 ng/mL) treatment. mRNA levels are given as fold increase or decrease over untreated control. Bars represent the means of 3 (microarray) and 5 (qPCR) independent experiments (±SEM). Significant differences to untreated control were calculated by ANOVA and Dunnett's post hoc test (* p≤0.05; ** p≤0.01).

Next we analysed a fraction of annotated genes that was generally regulated independent of the application route and DON concentration. The number of genes found in this group of the microarray analysis (25 genes) included 6 generally down-regulated and 19 up-regulated genes. The genes (summarised in Table 2) did not necessarily show a common functional background, however, two genes were related to ribosomal structures (RPS4X and RPS9). The findings of the microarray were further validated by the quantitative mRNA analysis of selected genes (qPCR). We selected two genes of the generally regulated group for qPCR validation, caveolin2 (CAV2) and endoplasmin precursor GRP94 (TRA1). CAV2 mRNA was generally up-regulated whereas TRA1 mRNA was generally down-regulated in the microarray. The results of qPCR confirmed the significant down-regulation of TRA1 as suggested by microarray except for the down-regulation found with 200 ng/mL DON from apical side (Fig. 4A). The general up-regulation of CAV2 found in microarray could not be confirmed in qPCR except for the 200 ng/mL apical DON application (Fig. 4B).

**Table 2:** Generally regulated genes found in all treatments investigated independent of applied DON concentration and application route.

Human Affymetrix ID	Accession Number	Gene Name	Product up regulated
202587_s_at	AJ604863	AK1	Adenylate kinase isoenzyme 1 (EC 2.7.4.3) (ATP-AMP transphosphorylase) (AK1) (Myokinase).
203324_s_at	CO990376	CAV2	Caveolin-2.
221021_s_at	CK462484	CTNNBL1	Beta-catenin-like protein 1 (Nuclear associated protein) (NAP) (NYD- SP19) (PP8304).
231769_at	CN163641	FBXO6	F-box only protein 6 (F-box/G-domain protein 2).
204145_at	AJ655733	FRG1	FRG1 protein (FSHD region gene 1 protein).
214094_at	CN154180	FUBP1	Far upstream element binding protein 1 (FUSE binding protein 1) (FBP) (DNA helicase V) (HDH V).
212642_s_at	BX919005	HIVEP2	Human immunodeficiency virus type I enhancer-binding protein 2 (HIV- EP2).
203202_at	CN164094	HRB2	HIV-1 Rev binding protein 2 (Rev interacting protein 1) (Rip-1). [Source:Uniprot/SWISSPROT;Acc:Q13601]
213067_at	L29130.1	MYH10	Myosin heavy chain, nonmuscle type B (Nonmuscle myosin heavy chain-B) (NMMHC-B).
1554747_a_at	BI183782	NEDD5	Septin 2 (NEDD5 protein homolog). [Source:Uniprot/SWISSPROT;Acc:Q15019]
201803_at	AF265351.1	POLR2B	DNA-directed RNA polymerase II 140 kDa polypeptide (EC 2.7.7.6) (RNA polymerase II subunit 2) (RPB2).
206789_s_at	NM_214264.1	POU2F1	POU domain, class 2, transcription factor 1 (Octamer-binding transcription factor 1) (Oct-1) (OTF-1) (NF-A1).
210307_s_at	CO953600	Q9H0H3	BTB/POZ KELCH domain protein
209444_at	BP457832	RAP1GDS1	Rap1 GTPase-GDP dissociation stimulator 1 (SMG P21 stimulatory GDP/GTP exchange protein)
219613_s_at	CO994328	SIRT6	NAD-dependent deacetylase sirtuin 6 (EC 3.5.1.-) (SIR2-like protein 6).
228254_at	AJ663381	STAM2	signal transducing adaptor molecule 2; STAM-like protein containing SH3 and ITAM domains 2.
202495_at	BG382185	TBCC	Tubulin-specific chaperone C (Tubulin-folding cofactor C) (CFC).
208942_s_at	CN162552	TLOC1	translocation protein 1; Dtrp1 protein; membrane protein SEC62, S.cerevisiae, homolog of [Homo sapiens].
34689_at	CF367877	TREX1	ATR-interacting protein (ATM and Rad3 related interacting protein).

Human Affymetrix ID	Accession Number	Gene Name	Product down regulated
207452_s_at	CO939543	CNTN5	contactin 5 isoform long; neural adhesion molecule [Homo sapiens].
219520_s_at	CN166871	Q9ULE0	
202845_s_at	BX924307	RALBP1	RalA binding protein 1 (RalBP1) (Ral interacting protein 1) (76-kDa Ral-interacting protein) (DNP-SG ATPase).
200933_x_at	CK461703	RPS4X	ribosomal protein S4, X-linked X isoform; 40S ribosomal protein S4, X isoform; cell cycle gene 2.
214317_x_at	CF365984	RPS9	40S ribosomal protein S9.
200599_s_at	BM190021	TRA1	Endoplasmin precursor (94 kDa glucose-regulated protein) (GRP94) (gp96 homolog) (Tumor rejection antigen 1).

In the next step we analysed annotated genes identified in response to various DON concentrations and application routes. We analysed the functional background of the regulated genes by functional clustering using the DAVID approach. The analysis included significantly regulated genes with human annotation. The analysis was separately done for the corresponding up- or down-regulated gene set and biological pathways were identified according to the KEGG database. Disease specific pathways were not considered. The most relevant functional clusters were summarised in Table 3. The four treatment groups exhibited a complex pattern of up- and down-regulated genes related to a broad range of pathways. As found in the initial analysis of single genes, the functional cluster involved in the generation of the protein proportions of ribosomes was down-regulated in all applied conditions. In contrast, genes of the spliceosome were up-regulated in all treatments. Besides these both, we could not find another pathway which was equally affected by all four treatments investigated. Commonly regulated genes were predominantly found in response to basolateral application of 200 and apical application of 2000 ng/mL DON. The regulated genes are involved in pathways of oxidative phosphorylation, ubiquitin mediated proteolysis, proteasomes, RNA degradation, focal adhesion and tight junctions. However, basolateral application of 2000 ng/mL DON exhibited a different pattern of involved pathways except for the shared pathways of ribosomes and spliceosomes. Only genes involved in endocytosis and focal adhesion were up-regulated in 2000 ng/mL DON applied from apical and basolateral side.

**Table 3:** Functional affiliation of significantly up- or down-regulated annotated genes in response to apical or basolateral DON treatment.

KEGG-Pathways			200 ap	200bl	2000 ap	2000bl
Metabolism	Carbohydrate metabolism	Citrate cycle (TCA cycle)		**+**		**+**
		Pentose phosphate pathway			**−**	
		Fructose and mannose metabolism			**−**	
	Energy metabolism	Oxidative phosphorylation		**+**	**+**	
	Nucleotide metabolism	Pyrimidine metabolism				**+**
	Amino acid metabolism	Arginine and proline metabolism	**+**			
		Glutathione metabolism				**−**
	Glycan biosynthesis and metabolism	N-glycan biosynthesis				**−**
		Glycosaminoglycan biosynthesis				**−**
		Glycosaminoglycan degradation		**−**		
	Metabolism of cofactors and vitamines	Folate biosynthese		**−**		**−**
Genetic information processing	Transcription	RNA polymerase			**+**	
		Basal transcription factors				**+**
		Spliceosome	**+**	**+**	**+**	**+**
	Translation	Ribosom	**−**	**−**	**−**	**−**
		Aminoacyl-tRNA biosynthesis				**+**
	Folding, sorting and degradation	Protein export				**+**
		SNARE interactions in vesicular transport				**+**
		Ubiquitin mediated proteolysis		**+**	**+**	
		Proteasome		**+**	**+**	
		RNA degradation		**+**	**+**	
	Replication and repair	DNA-replication		**−**		
Environmental information processing	Signal transduction	ErbB signalling pathway				**+**
		Wnt signalling pathway				**+**
		TGF-beta signalling pathway	**+**			
		Jak-STAT signalling pathway	**+**			
	Signalling molecules and interaction	ECM-receptor interaction				**−**
Cellular processes	Transport and catabolism	Endocytosis			**+**	**+**
		Lysosome		**−**		**−**
	Cell motility	Regulation of actin cytoskeleton		**+**		
	Cell growth and death	Cell cycle				**−**
	Cell communication	Focal adhesion		**+**	**+**	**+**
		Adherens junction			**+**	
		Tight junction		**+**	**+**	
		Gap junction				**−**

Significantly changed genes of the microarray were analysed with the database DAVID and changed KEGG pathways are shown for the four treatment groups. Up-regulated functions (+) and down-regulated genes (−) are indicated.

**Figure 5:**
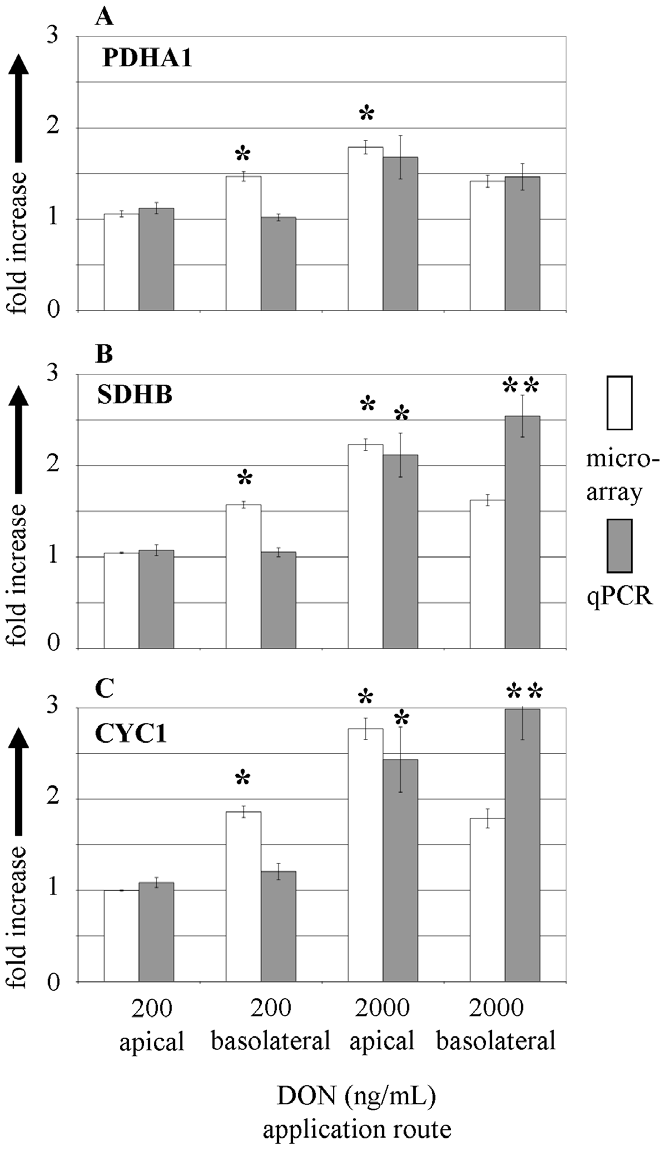
Expression of selected metabolic genes measured by microarray and qPCR in membrane cultured IPEC-J2 cells in response to apical and basolateral DON application. Regulation of transcripts of (**A**) PDHA1, (**B**) SDHB and (**C**) CYC1 in response to apical and basolateral DON (200 and 2000 ng/mL) treatment. mRNA levels are given as fold increase over untreated control. Bars represent means of 3 (microarray) and 5 (qPCR) independent experiments (±SEM). Significant differences to untreated control were calculated by ANOVA and Dunnett's post hoc test (* p≤0.05; ** p≤0.01).

**Figure 6:**
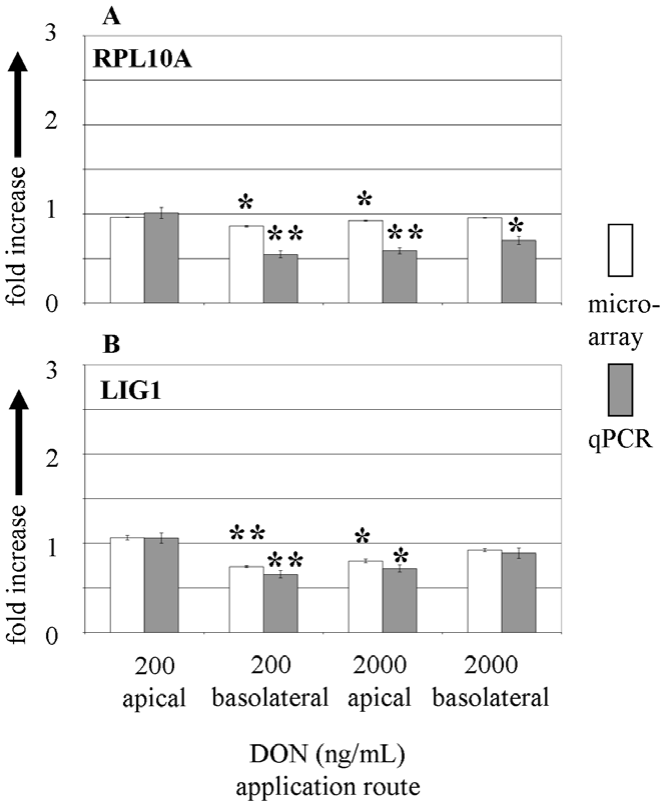
Expression of selected genes of the genetic information flow measured by microarray and qPCR in membrane cultured IPEC-J2 cells in response to apical and basolateral DON application. Regulation of transcripts of (**A**) RPL10A and (**B**) LIG1 in response to apical and basolateral DON (200 and 2000 ng/mL) treatment. mRNA levels are given as fold increase over untreated control. Bars represent means of 3 (microarray) and 5 (qPCR) independent experiments (±SEM). Significant differences to untreated control were calculated by ANOVA and Dunnett's post hoc test (* p≤0.05; ** p≤0.01).

**Figure 7:**
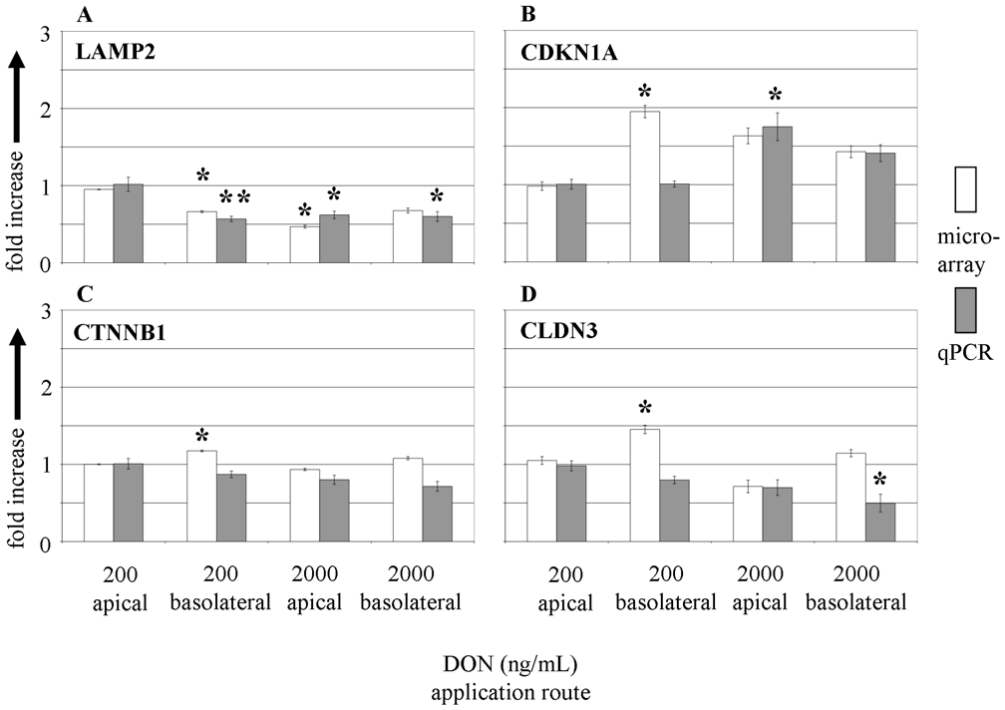
Expression of selected genes of cellular processes by microarray and qPCR in membrane cultured IPEC-J2 cells in response to apical and basolateral DON application. Regulation of transcripts of (**A**) LAMP2 (lysosomes), (**B**) CDKN1A (cell cycle), (**C**) CTNNB1 (focal adhesion) and (**D**) CLDN3 (tight junction) in response to apical and basolateral DON (200 and 2000 ng/mL) treatment. The mRNA levels are given as fold increase over untreated control. Bars represent means of 3 (microarray) and 5 (qPCR) independent experiments (±SEM). Significant differences to untreated control were calculated by ANOVA and Dunnett's post test (* p≤0.05; ** p≤0.01).

Genes for qPCR verification were selected to cover a broad range of significantly affected mechanisms including metabolism, genetic information processing and cellular processes as identified by microarray. The corresponding gene names and associated pathways are summarised in Table 4. The quantitative results of the microarray and qPCR are given in Fig. 5 (metabolism), Fig. 6 (genetic information procession) and Fig. 7 (cellular processes). Essentially, both methods indicated similar trends regarding the up- or down-regulation of the analysed mRNA, however, differences between both methods were found. In Fig. 5 the expected increase in transcription of PDHA1, SDHB and CYC1 as found in microarray with 200 ng/mL DON basolateral was not confirmed with qPCR. Significantly increased transcript levels of SDHB and CYC1 were found in response to 2000 ng/mL DON apical with microarray and qPCR. The application of 2000 ng/mL DON from basolateral triggered massive destruction of epithelial cell layer as detected by TEER (Fig. 1), however, all three genes of the cellular energy generation were numerically or significantly increased in comparison to untreated control.

**Table 4:** Selected genes for qPCR analysis and their relation to functional pathways.

KEGG-pathways			Protein	Gene
Metabolism	Carbohydrate metabolism	Citrate cycle (TCA)	Pyruvate dehydrogenase	PDHA1
	Energy metabolism	Oxidative phosphorylation	Succinate dehydrogenase	SDHB
			Cytochrome C-1	CYC1
Genetic information processing	Translation	Ribosome	Ribosomal protein L10a (60S subunit)	RPL10A
	Folding, sorting and degradation	Protein processing in endoplasmic reticulum	Endoplasmin precursor (GRP94)	TRA1
	Replication and repair	DNA replication	DNA ligase 1	LIG1
Cellular processes	Transport and catabolism	Endocytosis	Caveolin-2	CAV2
		Lysosome	Lysosome-associated membrane glycoprotein 2	LAMP2
	Cell growth and death	Cell cycle	Cyclin-dependent kinase inhibitor 1	CDKN1A
	Cell communication	Focal adhesion	ß-Catenin	CTNNB1
		Tight junction	Claudin-3	CLDN3

Grouping of functional pathways was done according to the KEGG database.

In the next step we analysed in detail two genes related to the cellular information flow, namely genes of the ribosomal protein L10a (RPL10a) and DNA ligase 1 (LIG1) as shown in Fig. 6. Both genes exhibited significantly reduced transcription levels with 200 ng/mL DON basolateral, but not with 200 ng/mL apical application. A comparable reduction was reached by the 10 times higher application of 2000 ng/mL from apical side. Interestingly, the effect of DON on the selected genes was reduced in the 2000 ng/mL basolateral condition.

Finally, a set of selected genes related to lysosomal function (LAMP2), cell cycle (CDKN1A), focal adhesion (CTNNB1) and tight junction (CLDN3) was analysed and is shown in Fig. 7. All genes exhibited significant regulation in microarray when treated basolaterally with 200 ng/mL DON, but did not respond to the same concentration applied from apical. The significant down-regulation of LAMP2 as found in microarray was confirmed, however, the up-regulation in CDKN1A, CTNNB1 and CLDN3 as suggested by microarray could not be verified in qPCR. Significant effects of side-specific DON application were detected with qPCR after apical application of 2000 ng/mL DON (CDKN1A, up-regulation) and basolateral application of 2000 ng/mL DON (CLDN3, down-regulation). In microarray analysis these conditions did not significantly regulate transcription.

## Discussion

Membrane cultures of IPEC-J2 cells represent a valuable model of the epithelial barrier *in vitro* exhibiting essential features of the *in vivo* situation [3]. Typically tight junction components like ZO-1 and claudin-3 can be detected in the cultured monolayer [4]. In previous studies we have shown that the toxicological potency of the mycotoxin DON is essentially dependent on the spatial distribution of the mycotoxin. The cell culture model was found to be insensitive to apical application whereas basolateral application of same concentration elicited massive cellular damage [4]. Here we analysed the molecular basis of DON toxicity and the increased sensitivity to basolateral challenge. Two dosages, 200 and 2000 ng/mL, were selected, reflecting reported intestinal DON concentrations *in vivo* [12]. The 200 ng/mL dosage was essentially non-toxic in conventional cell culture assays [8]. A similar non-toxic threshold of 100 ng/mL was found by Vandenbroucke et al. in conventionally cultured 1 d old IPEC-J2 cells, however, in 21 d old cultures the threshold was above 10 µg/mL during 24 h incubation [13]. This situation is comparable to the apical application of DON in the IPEC-J2 membrane cultures as shown in our previous study [4].

We first analysed the general mechanisms involved in DON toxicity on epithelial cells for all applied concentrations and spatial orientations, as indeed only a limited number of genes was significantly regulated in all treatments in the microarray analysis. As shown in Table 2 the genes of this group did not exhibit a functional clustering, however, two genes (RPS4X and RPS9) related to ribosomal structure were down-regulated. This finding is in line with previous findings as DON is known to trigger ribotoxic stress [14], [15]. We have further analysed the gene TRA1 (Glucose regulated protein 94, GRP94, Endoplasmin) by qPCR (Fig. 4A). We found a significant qPCR validated reduction of the mRNA level of this gene which is known to act as a Ca^2+^ buffering chaperone in the endoplasmic reticulum (ER) and is involved in the ER stress response [16], [17]. The effect of DON on ER triggering a stress response was investigated in a T-cell model [18] and in mouse peritoneal macrophages [19]. The authors analysed the effect of DON on another glucose regulated protein also involved in ER stress response (GRP78, BiP) on protein and mRNA level. In the T-cell model a reduction of the mRNA of GRP78 was reported, but not in the macrophage model. The regulation of TRA1 was not analysed in those experiments. In our array data we could not detect a significant regulation of GRP78 independent of the experimental condition. It has been shown that induction of GRP78 is involved in DON toxicity, likely via NFkappaB activation [18]. The activation of NFkappaB in response to ER stress is known, however, the role of TRA1 remains to be elucidated [20].

The microarray data obtained in response to apical or basolateral application were analysed by DAVID. In Table 3 the functional clustering and biochemical pathways involved in DON toxicity are summarised. The analysis gave further indications for the ribotoxic effects of DON. It is conspicuous that only two of the investigated mechanisms, ribosomal and spliceosomal pathway, were regulated in all conditions tested. The regulation of the ribosomal pathway is consistent with the idea that DON acts initially on the ribosomal structure as reported by other groups before [21]. The effect of DON on mRNA of ribosomal proteins is in line with these findings and confirms the validity of our investigation.

In contrast to the reduced expression of ribosomal related genes, an increase of genes of the spliceosomes was found in all conditions. As the spliceosomal processing precedes the ribosomal translation it could be interpreted as a defence response of the cell to cope with the DON mediated failure of ribosomal transcription and protein generation [22].

The microarray data indicated an enhanced expression of genes related to the cellular energy metabolism. Despite the fact that the selected key genes of the tricarboxylic acid cycle (TCA cycle) and mitochondrial energy metabolism did not exhibit an increase with 200 ng/mL DON basolateral in qPCR the result of the DAVID analysis generally indicated the need of enhanced energy generation in response to DON challenge. Apical application of 2000 ng/mL DON suggests also a cellular response to enhanced energy requirement as evidenced by qPCR (Table 3). The increase of genes related to the oxidative phosphorylation also confirms the role of oxidative stress in DON toxicity [23]. Oxidative stress has been reported for other mycotoxins like citrinin [24]. Averufin from *Aspergillus versicolor* acts as an uncoupler of the oxidative phosphorylation in rat liver mitochondria [25]. As shown in a cell culture model of HL-60 (human promyelocytic leukemia cells), patulin, a secondary metabolite of various *Penicillium* and *Aspergillus* species, triggers intracellular generation of reactive oxygen species (ROS) detected by oxidation of dihydroethidium [26]. Interestingly, an increase in glutathione-S-transferase gene was reported in this investigation analysing the citrinin toxicology in yeast, which likely acts as a detoxification mechanism [24], [27]. In contrast to these results we found a general decrease of the glutathione metabolism and the glutathione-S-transferase gene (Table 3).

In previous experiments we found an effect of DON on cell cycle of IPEC-J2 cells after 72 h of incubation [8]. In this context we analysed the expression of CDKN1A (cyclin dependent kinase inhibitor 1A, p21, p21Cip/WAF1). Yang et al. have been shown that the cell cycle arrest is mediated by stabilisation of CDKN1A mRNA [15]. Here we found significantly increased mRNA levels of CDKN1A in response to 200 ng/mL basolateral (microarray) and 2000 ng/mL apical (qPCR) DON treatment. Interestingly, in a recent article of Owusu-Ansah et al. [28] a signalling pathway was described in *Drosophila melanogaster* connecting ROS stress of mitochondria with the G1-S cell cycle checkpoint. The *Drosophila* analogue of CDKN1A, dap, was involved in this retrograde signalling.

In contrast to the genes of the cellular energy metabolism, regulated predominately at the high (2000 ng/mL apical or basolateral) DON concentration, the investigated genes of the cellular information flow responded clearly to the low (200 ng/mL) basolateral DON concentration. This finding supports the view that DON acts initially on the information process and that the increase of energy metabolism is a response of the cell to cope with the DON challenge. The interpretation of the results of the 2000 ng/mL basolateral challenge regarding the cellular information flow is difficult as the cell layer was already severely disturbed.

We found in our microarray dataset a significant decrease of the major lysosomal membrane protein LAMP-2 in response to basolateral application of the lower (200 ng/mL) DON challenge whereas the apical application was without effect. This result was confirmed in the qPCR measurement. Furthermore, it has been described that DON adversely affects the lysosomal function when applied in low micromolar concentrations using a neutral red uptake assay in Caco-2 cells [23]. In a conventional cell culture approach we found a toxic effect of DON 2000 ng/mL after 24 to 72 h in IPEC-J2 cells [8] but not at 200 ng/mL. Similarly, in membrane cultures we found a constant transcription of LAMP-2 after 72 h of 200 ng/mL application from apical. In contrast, a significant confirmed reduction of transcription was found in response to 200 ng/mL basolateral application. The functional role of LAMP-2 in DON toxicity is unknown. LAMP-2 was proposed as a regulator for selective uptake and degradation of cytosolic proteins by lysosomes [29], [30].

The technique of microarray analysis is a powerful tool for analysing the effects of mycotoxins on epithelial cell culture systems. It is essential that the analysis covers a large array of functional mechanisms on a mRNA level, which can be related to many data already described in the literature. This confirmation of data considerably enhances the value of new findings. Extrapolating the *in vitro* results to the *in vivo* situation we suggest that DON, once taken up in the blood stream, can act on the basolateral compartment as a strong modulator of epithelial cellular physiology.
